# Enhancing grid resiliency in distributed energy systems through a comprehensive review and comparative analysis of islanding detection methods

**DOI:** 10.1038/s41598-024-62690-z

**Published:** 2024-05-27

**Authors:** Mangesh S Kulkarni, Sachin Mishra, Suresh Kumar Sudabattula, Naveen Kumar Sharma, D. Baba Basha, Mohit Bajaj, Milkias Berhanu Tuka

**Affiliations:** 1 Sharad Institute of Technology College of Engineering, Yadrav, Maharashtra India; 2 School of Electronics and Electrical Engineering, Lovely Professional University, Phagwara, Punjab India; 3 Electrical Engineering Department, I. K. G. Punjab Technical University, Jalandhar, 144603 India; 4 Department of Information Systems, College of Computer and Information Sciences, Majmaah University, 11952 Al-Majmaah, Saudi Arabia; 5 Department of Electrical Engineering, Graphic Era (Deemed to be University), Dehradun, 248002 India; 6 Hourani Center for Applied Scientific Research, Al-Ahliyya Amman University, Amman, Jordan; 7 Graphic Era Hill University, Dehradun, 248002 India; 8 Department of Electrical and Computer Engineering, Addis Ababa Science and Technology University, Addis Ababa, Ethiopia

**Keywords:** Distributed generation, Signal processing, Islanding detection, Microgrid, Non-detection zone, Artificial neural network, Renewable energy, Energy science and technology, Engineering

## Abstract

Reduction of fossil fuel usage, clean energy supply, and dependability are all major benefits of integrating distributed energy resources (DER) with electrical utility grid (UG). Nevertheless, there are difficulties with this integration, most notably accidental islanding that puts worker and equipment safety at risk. Islanding detection methods (IDMs) play a critical role in resolving this problem. All IDMs are thoroughly evaluated in this work, which divides them into two categories: local approaches that rely on distributed generation (DG) side monitoring and remote approaches that make use of communication infrastructure. The study offers a comparative evaluation to help choose the most efficient and applicable IDM, supporting well-informed decision-making for the safe and dependable operation of distributed energy systems within electrical distribution networks. IDMs are evaluated based on NDZ outcomes, detection duration, power quality impact, multi-DG operation, suitability, X/R ratio reliance, and efficient functioning.

## Introduction

The increasing penetration of DG based on renewable resources into the UG is primarily due to the growing emphasis on sustainability around the world. DERs present a range of electrical safety challenges for power systems engineers and researchers, notwithstanding their significant economic potential. Important factors that require attention include phase imbalances, voltage fluctuations, short circuits, deterioration of PQ, and, most importantly, the primary topic of this study: Islanding^1^.

**Figure 1:**
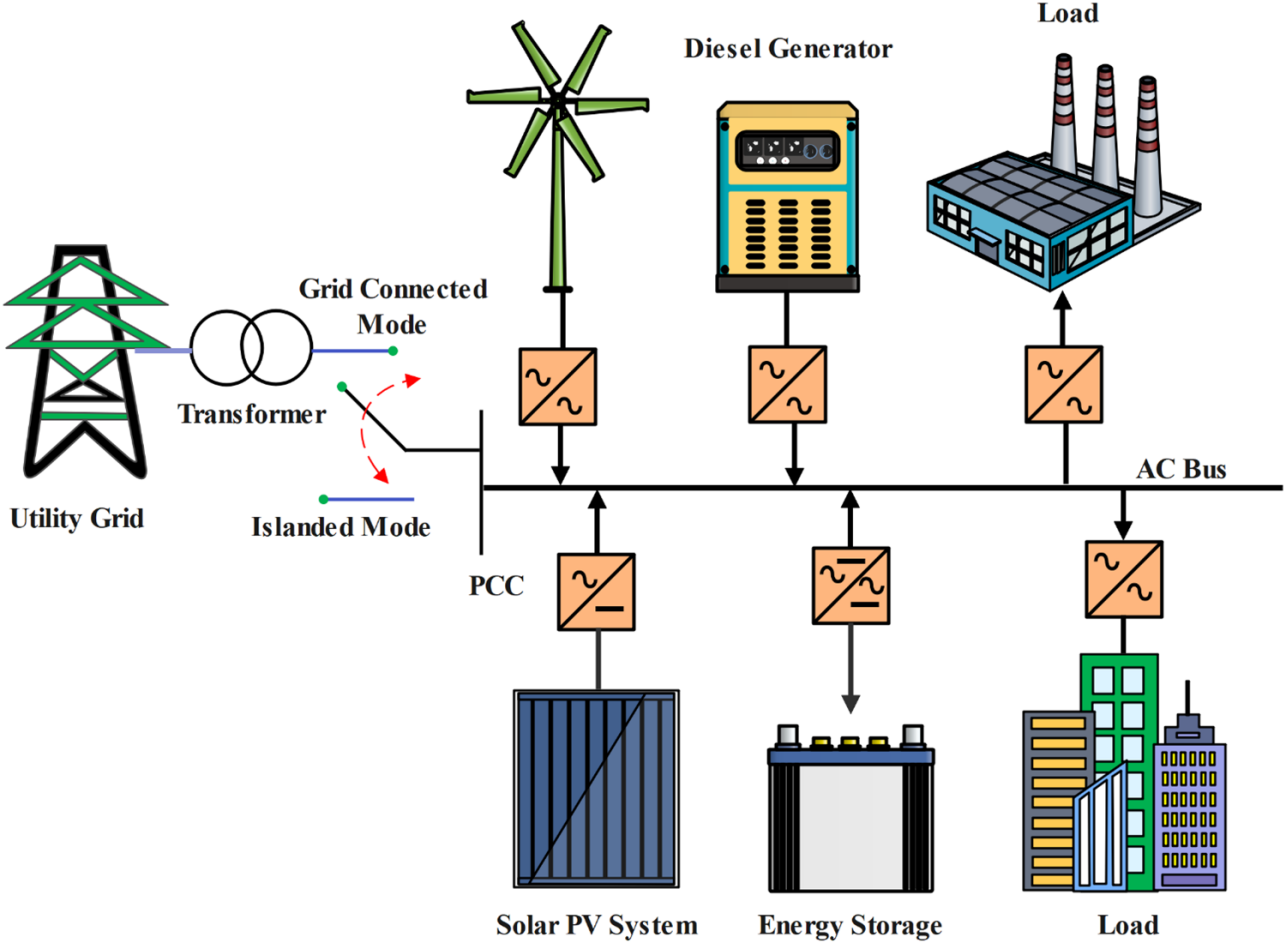
Microgrid operations—grid-connected and islanded modes.

IS is the practice of DG Systems continuing to operate electrically even in the event of a disconnection to the UG^2^ as shown in Fig. 1. This could happen unintentionally or on intentionally. Remarkably, intentional IS is a useful way to provide power to remote locations or to maintain power in affected areas. On the other hand, unintentional IS presents several hazards^3^, such as:

1. *Danger to personal safety*: workers performing maintenance can erroneously believe that power lines are turned off and carry out tasks without taking the necessary precautions.
2. *Hazard of excessive voltage and frequency*: In the event of a UG outage, voltage and frequency may exceed allowable limits, which could cause damage to equipment.
3. *Possible out-of-phase reconnection*: This could result in significant transient torques applied to rotating machinery, such as synchronous generators.
4. PQ degradation as a result of interactions between local load impedances and the output inverter current’s harmonic content.

**Figure 2:**
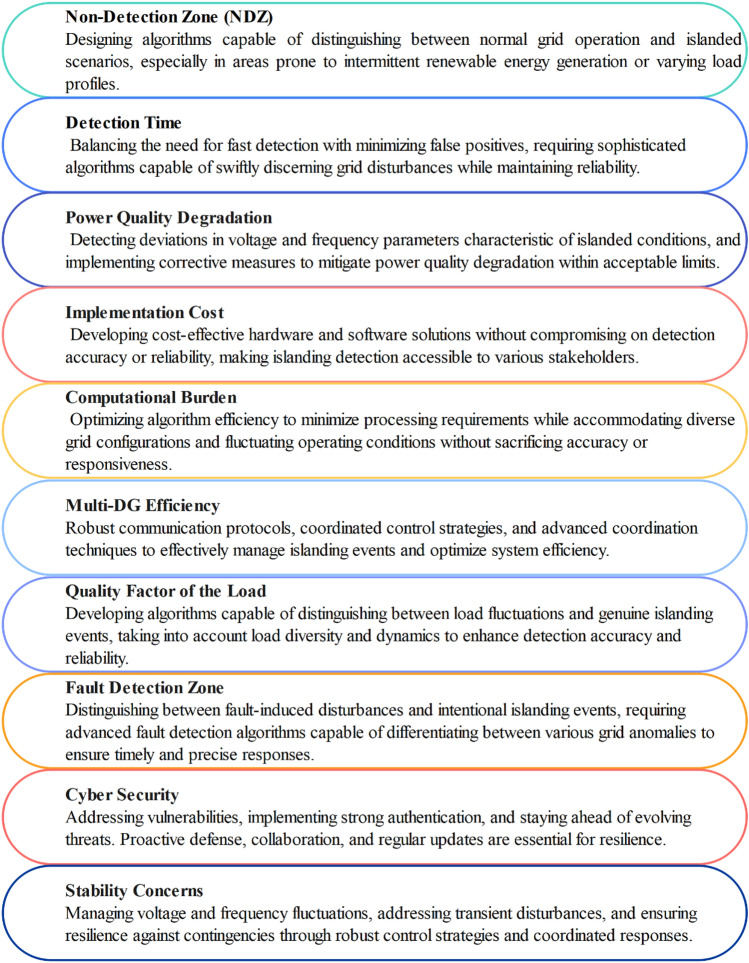
Key challenges for challenges for islanding detection.

Intentional grid shutdowns carried out for maintenance procedures, electrical malfunctions, human error, or sabotage are the primary sources of the unintentional IS phenomenon^4^. Reducing the NDZ where islanded conditions might go undetected^5,6^, obtaining quick T_D_ to stop extended islanding events^7^, preserving power quality in the face of grid disruptions , striking a balance between implementation costs^8^ and efficacy, controlling computational loads for real-time operation^9^, coordinating detection across multiple DG sources^10–12^, taking load characteristic variability into account, and reliably differentiating between fault conditions, fault detection zone^13,14^ and intentional islanding events are the main challenges related to islanding detection as shown in Fig. 2.

To provide prompt and dependable IS detection following the formation of the IS state, IDMs can be categorized based on their location and principle of operation. IDMs are classified as remote or local based on their location. The NDZ issue is resolved by remote methods. When remote solutions rely on advanced communication technology, they run the risk of being expensive and difficult to execute, even with their accuracy^15^. Impedance Insertion^16^, PLCC^17^, Transfer-Trip Schemes^18^, PMU^19^, MPMU^20^, and SCADA^21^ are some of the primary implementations of these strategies. Conversely, local IDMs are located on DG side. Within MGs, local approaches are frequently used because they are inexpensive to adopt. Large NDZ and false trips are common with local IDMs, even with their increased popularity. However, by using mathematical or computational methods such as ML algorithms and positive voltage or frequency feedback, these issues can be reduced and even completely removed. This illustrates how the high cost of remote IDMs contributes to their dependability. On the other hand, local methods are classified into three: passive, active, and hybrid, methods. Phase shift^22,23^, voltage^22^, frequency^24^, ROCOF^25^, and total or individual HD^26^ are among the electrical parameters that passive methods directly monitor at the PCC^27^. When it comes to PQ evaluation, the main benefit of passive IDMs is that they are non-intrusive. Low precision and a large NDZ are potential drawbacks, though. Conversely, active methods entail introducing small perturbations into specific inverter operation parameters. The primary objective is to set up conditions that lead to the inverter losing stability following a UG loss, enabling it to function beyond the constraints established by global standards^28^. This category includes techniques such as harmonic insertion, high-frequency signal injection, and frequency and voltage drift approaches. Maintaining the balance between the impact on PQ and efficacy is necessary for active IS detection.

Combining the best features of both active and passive IDMs, hybrid IDMs function. If the control system identifies an IS scenario, it will introduce an active perturbation into the system for a predetermined period. SFS techniques^29^, Voltage imbalance and frequency set point^30^, ROCOF techniques^31^, Voltage and real power shift^32^, and SMS techniques are the primary examples of this type of solution. Comparing hybrid systems to passive approaches, their NDZ will be less, but compared to active solutions, it will be greater. The use of ANNs^33^, DL^34^, DT^35^, FL^36^, SVM^37^, and ANFIS^38^ have drawn the attention of many researchers in recent years. These methods have the significant benefit of not requiring communication channels. Furthermore, there is no interference with PQ or efficiency. Nevertheless, depending on the volume of inputs into the algorithm, these methods require a significant computing load and a high number of sensors. Lastly, there is yet another class of IDMs based on passive detection that enhances robustness, dependability, and efficiency through the use of SP techniques. These include the following: Mathematical Morphology (MM)^39^, ST^40^, FT^41^, WT^42^, KF^43^, VMD^44^, and EMD^45^.

This setting reveals that the development of IDMs has attracted the interest of the scientific community, and many authors have been actively involved in the production of novel and remarkably effective methods. A lot of time and energy has been put in by numerous researchers to share their discoveries with the scientific community and deepen their comprehension through works that present the most advanced IDMs available today.

Despite the advantages of each effort, the substantial research gap on IDMs cannot be sufficiently addressed by them due to the unique nature of their approaches. It also includes a chronology of the primary techniques' development, encompassing the most current developments from the specialized technical literature as discussed in Table 1.

**Table 1:** Comparison taxonomy of current survey.

Reference	Contributions	Shortcomings	Unique contributions of the current survey
^46^	Islanding detection strategies based on ML/SP technologies were discussed	Does not cover the historical progression of the active methods and does not provide a deeper analysis of the remote methods	This review paper uniquely addresses the significant gap in the existing literature by offering a comprehensive consolidation of regulatory requirements and identifying the key challenges of IDMs face for functionality. It further presents a detailed timeline of the evolution of main methods, integrating the latest contributions from specialized technical literature. Additionally, the paper conducts an in-depth comparative analysis of IDMs based on critical criteria such as Non-Detection Zone (NDZ), Detection Time, compatibility with multiple distributed generators (DGs), suitability for various microgrid configurations (SSSG / SGBMG / IBMG), dependence on X/R ratio, effectiveness in weak grid/strong grid scenarios, and power quality impact
^3^	Covers passive and active, hybrid and remote islanding detection strategies. This paper presents one of the first studies on smart IDM, providing a comprehensive assessment of their strengths and weaknesses	Vital passive techniques like voltage imbalance and harmonic detection were not examined. Furthermore, it does not explore recent advances stated in the literature about hybrid and active IDMs, nor does it fully cover ML and SP techniques. ignores remote PMU and Micro PMU-based approaches and does not adequately examine current developments in passive and active methods	
^47^	An extensive analysis of hybrid, active, and passive methods is given. This paper's primary strength is its comparison of the NDZ of passive methods to important network coding requirements. Additionally, it provides a thorough analysis of the development of passive and active IDMs, and methods used to overcome their limitations	does not address remote methods as well as smart and SP methods	
^48^	a systematic review of intelligence-based IDMs is provided. In order to extract characteristics from the discovered data, the analysis takes into account the primary intelligent classifiers and technologies	Does not address IDMs other than intelligence-based IDMs	
^49^	a comprehensive review of IDMs based on signal processing is performed	Does not address IDMs other than signal-processing-based IDMs	
^50^	outlined the principal IDM patents awarded in the United States	Included only summary of patents granted in USA	
^51^	An extensive analysis is given of intelligent, hybrid, passive, active, and SP IDMs	Limited explanation of different IDMs, and does not cover recent developments in IDMs	
^52^	Comprehensive explanation of symmetrical real-time PMU data, showcasing various PMU data analysis methods, and outlining suggested plans for the IDMs	Does not address IDMs other than PMU data-based IDMs	

Hence, considering the literature information, this paper aims to support the scientific community. In “Standard Test Setup and Standards for ID” Section, we examine test methods and standards utilized for islanding detection, offering an overview of established protocols and regulatory frameworks that govern this pivotal aspect of MG operation. “Islanding Detection Methods” extensively investigates islanding detection methods, providing a thorough analysis of different techniques employed in the field. “Recommendations and Future Trends” Section builds upon this groundwork by presenting recommendations and insights into future trends in ID, highlighting emerging technologies, as well as challenges and opportunities for advancement. Our conclusion synthesizes the key findings and implications derived from our analysis, emphasizing the importance of robust islanding detection strategies in maintaining the stability and reliability of microgrid systems. Additionally, we conduct a detailed comparative analysis of various islanding detection methodologies, elucidating their strengths, weaknesses, and suitability across diverse operational developments.

## Standard test setup and standards for ID

The growing use of DG based on RES has many advantages for the economy, society, and environment, but it can also result in several problems related to electrical security and deterioration of PQ. In this perspective, the inverters commercialization and DG grid integration to the UG must follow standards-based rules.

The commercialization of DGs and its integration into the UG are contingent upon the provision of IS protection. The minimal specifications needed for IS Protection in order to commercialize inverters and link DG Systems to the UG are recommended by a number of Standards as per Table 2.

**Table 2:** ID standards recommendations.

Standard	Quality Factor	Detection Time	Voltage Range	Frequency Range
IEEE 1547	1	Less than 2 s	0.88–1.1 pu	59.3–60 Hz
IEEE 929–2000	2.5	Less than 2 s	0.88–1.1 pu	59.3–60.5 Hz
IEC 62,116	1	Less than 2 s	0.88–1.15 pu	59.3–60 Hz
UK G83/3 (17 kW/ph or 50 kW/three phase)	0.5	Less than 0.5 s	0.87–1.1 pu (stage 1) 0.8–1.19 pu (stage 2)	47.5–51.5 Hz (stage 1) 47–52 Hz (stage 2)
UK G83/2 (DGs up to 16 A/phase)	0.5	Less than 0.5 s	0.87–1.1 pu (stage 1) 0.8–1.19 pu (stage 2)	47.5–51.5 Hz (stage 1) 47–52 Hz (stage 2)
ERDF-NOI- RES 13E Japanese JIs	0	Between 0.5 and 1 s (passive method) Less than 2 s (active method)	Setting value Setting value	Setting value Setting value
Canadian C22.2 No. 107-01	2.5	Less than 2 s	0.88–1.06 pu	59.5–60.5 Hz
UL 1741	1	Less than 2 s	0.88–1.1 pu	59.3–60.5 Hz
Korean	1	Less than 0.5 s	0.88–1.1 pu	59.3–60 Hz
German VDE 0126-1-1	2	Less than 0.2 s	0.88–1.15 pu	47.5–50.2 Hz
French	2	Instantly	0.88–1.06 pu	49.5–50.5 Hz

**Figure 3:**
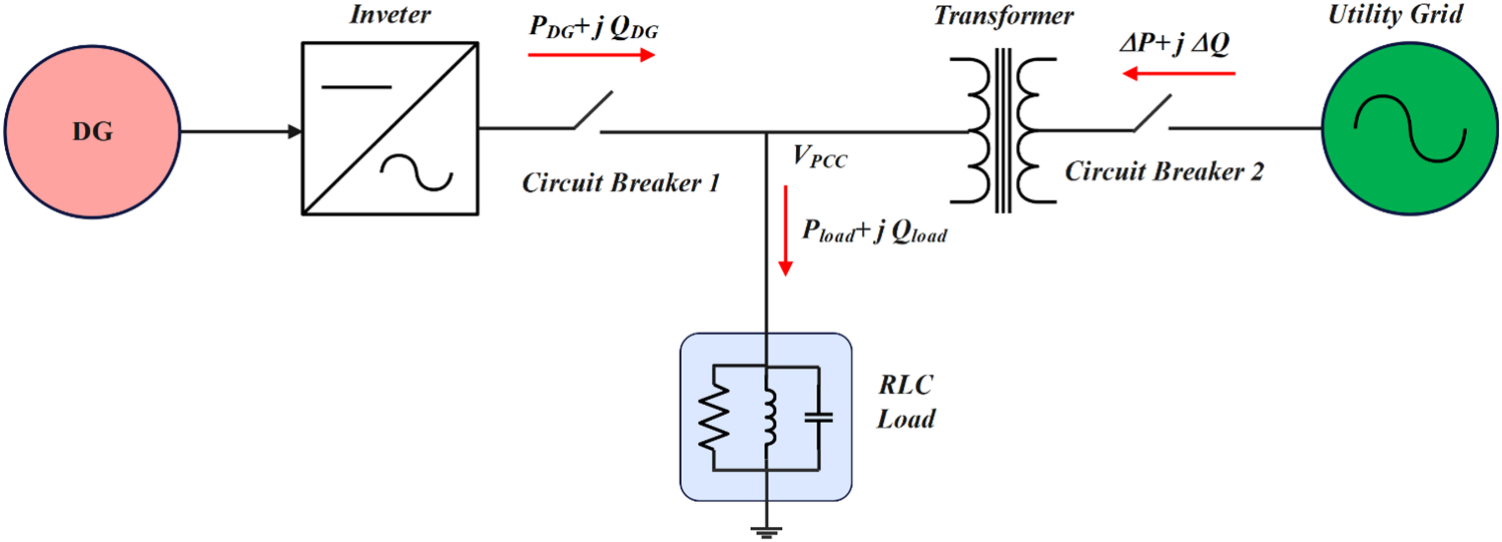
Standard setup for IDM test.

The setup for the IS detection test process is the same in all normative documents, despite their variances. The general configuration for an IDM test is shown in Fig. 3. The load's main goal is to reduce the UG's influence on the balance of the system. For this reason, to reduce the amount of active power that must be drawn from the UG, the resistive parameter must perfectly match the power rating of the inverter. To further lessen the UG's reactive power contribution, the LC pair should resonate at grid frequency. It is possible to conduct the test using an electrical load or a passive load^53,54^. Guidelines for performing IS detection tests on DG systems are available in several standards. A wide range of reactive load parameters should be used while repeating the IS detection test, according to standard protocol^2,55–57^.

## Islanding detection methods

### Remote techniques

**Figure 4:**
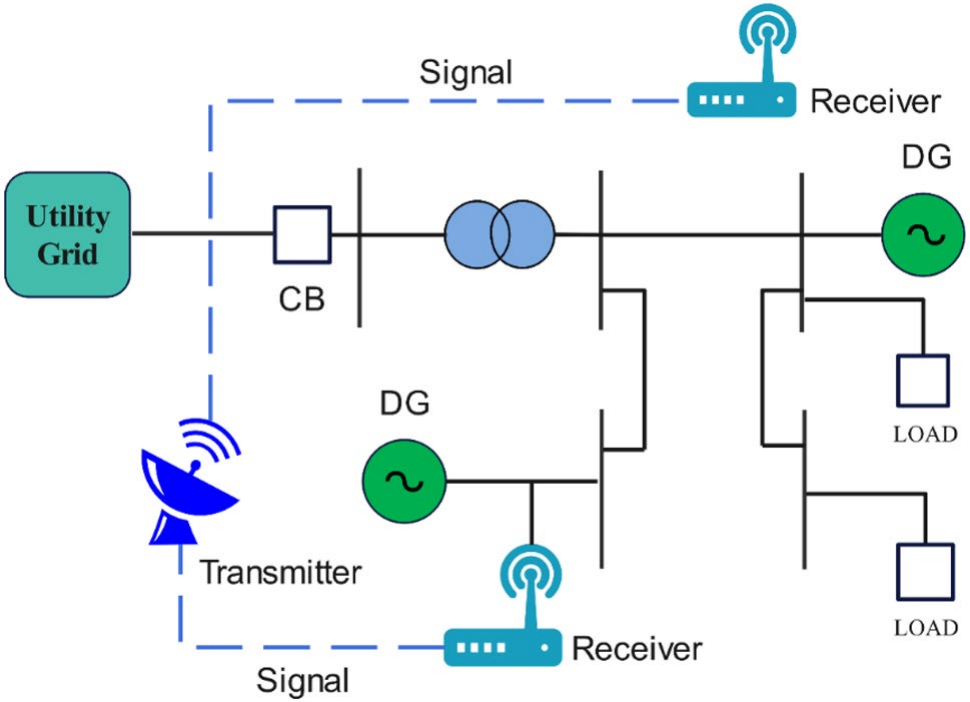
Remote IDM.

**Figure 5:**
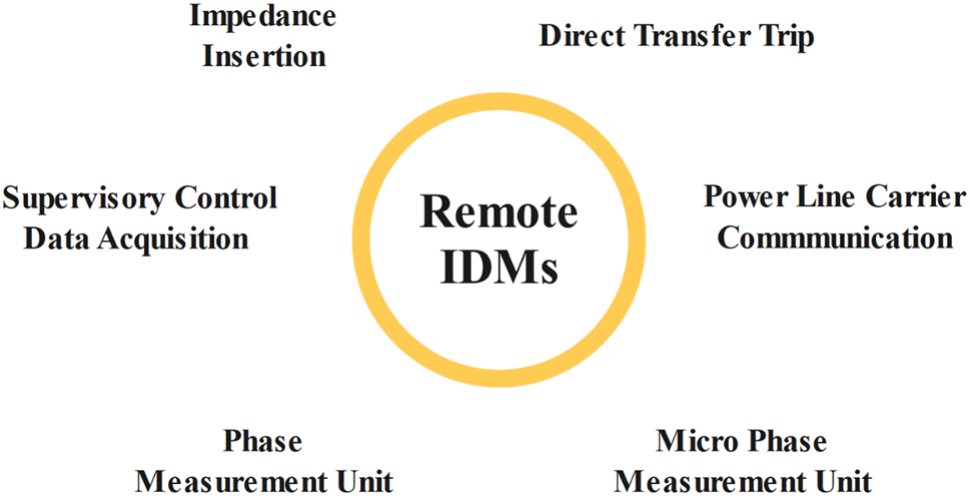
Remote IDMs.

Communication or SP technologies are used by remote or grid-resident IDMs to identify IS events. The use of remote IDMs is recommended because it can avoid several issues related to local IDMs, including reduced efficacy when there are several DG units, negative impacts on PQ, and the existence of NDZs. However, small and medium-sized DG systems cannot afford the high price and complicated implementation associated with remote IS detection. Figure 4 describes remote IDM working and Fig. 5 enlists different remote IDMs. ECS, DTT, and PLCC are a few well-known methods of remote techniques^15^.

#### Impedance insertion

The PCC voltage and frequency is altered beyond the allowable operating range by activating a specific impedance element, usually a bank of capacitors, using the Impedance Insertion method. This creates a reactive power imbalance. Nevertheless, this approach has several shortcomings, such as the expense for capacitor bank, sluggish reaction times, and the possibility of running into NDZs^16^. However, its wider use has been hindered by the need for an additional device attached to the PCC and the ensuing expensive implementation costs.

#### Direct transfer trip

The central control unit of an IDM based on DTT keeps track of all circuit breakers that can isolate the DGs and determines which areas are islanded^3^. This method has excellent effectiveness and precision, just like other remote IDM systems, however, it has drawbacks due to expensive costs and complicated implementation. This is due to its reliance on a supervisory system and communication infrastructure, which includes radio, dedicated fiber, and leased phone lines^18^. In a study published in^58^, three naturally occurring IS events were examined in a power plant with different generators that used the DTT strategy to prevent islanding. The analysis's conclusions suggest that to avoid having extended detection periods, the DTT technique should not be used in systems that can perform load shedding. Furthermore, the existence of reactive compensators may have a substantial effect on the scheme's performance.

#### Power line carrier communication

The power line infrastructure itself serves as the communication channel for a PLCC system. UG interruption detection happens if the IS signal stops during its four conduction cycles, which is how it is normally conveyed^59^. For communication lines longer than 15 km, repeaters are required for PLCC-based IDMs to prevent signal attenuation^15^. However, there haven't been many articles on this method in the literature because of its high complexity and implementation expense. Paper^17^ provided an overview of the original proposal for using PLCC for IS detection. In particular, for smaller generation systems, it emphasizes that high-frequency signals should be avoided due to their propensity to be attenuated by the series inductors found in distribution transformers. In addition, to reduce complexity, the information must be conveyed purposefully and slowly. To further reduce extra implementation costs, the study combines a low-cost receiver with a commercial automatic meter reading device. In addition, it lists several conditions that must be met for an IDM based on PLCC to be economically viable. A study that presents evaluation of a PLCC based IDM used for real DG configuration is presented in^57^. Numerous findings on the signal attenuation brought about by the transmission line’s inherent qualities and local load characteristics are drawn from this research. An analysis of the signal attenuation caused by a medium voltage transformer is presented in^60^, along with a mathematical equation that characterizes this attenuation. Finally, a sensitivity analysis of a PLCC-based IDM is carried out by^61^.

#### Supervisory control data acquisition method

This system is appropriate for IS detection since it manages a variety of circuit breakers, including those that link DG to the UG. This method has benefits and drawbacks that are comparable to those of remote approaches. As a result, it may remove NDZs without sacrificing PQ. However, SCADA-based IDMs^21^ are not practical for small or medium-sized DG systems because of their high cost, complexity, and need for remote operators^62^.

#### Phase measurement unit (PMU) method

PMU gives real-time information about the electrical phasor's phase and magnitude in the electrical system^63^. PMUs are used in many different aspects of power systems, including monitoring, synchronization, purposeful islanding, and transmission voltage level detection of inadvertent UG outages. About intentional IS operations^64^, carried out a thorough analysis of intentional IS. The results disclosed that the PMU device successfully and with little amplitude and phase error reconstructed the signals that were evaluated. Furthermore, the study found that when compared to SCADA systems, PMU devices provide a more reliable depiction of electrical parameter. On the other hand, PMU is also used for inadvertent IS detection. Two different PMU IDMs, the CADM and the FDM, are proposed in^65^. Whereas the latter takes advantage of the phase difference between two buses, the former compares the observed frequency with a predetermined threshold to identify loss of mains. The outcomes show that both approaches identify IS in three distinct circumstances with success. Additionally, it is seen that the FDM and CADM combination is sufficiently selective to avoid falsely tripping the DG during six non-IS incidents. A new IDM is presented in^66^, which consists of a PMU system that uses an intelligent tree algorithm to detect IS. Systematic Principal Component Analysis (SPCA) is used in^19^ to create the Synchro-phasor IDM, which performs well even when training data is updated^67^. Suggests a remote solution that combines PMU and SCADA techniques to produce accurate IS detection with no instances of misclassification. Additionally^68,69^, provide descriptions of other effective PMU-based accidental IS detection approaches.

#### Micro phase measurement unit (MPMU)

The MPMU was first proposed in^70^. These units are specifically designed to handle the special characteristics of distribution lines^71^. The two main aspects that are monitored by the IDM based on MPMU in^20^ are the Cumulative Sum of Frequency Difference and the Phase Angle Difference. This plan takes advantage of the phase's effect on frequency once sources are lost. IS event detection is achieved by using a Pearson correlation coefficient. Findings show that, the system can reliably identify IS in 0.25 s. Moreover, it can reliably detect non-IS scenarios and is robust against measurement noise. A unique IDM using four MPMU devices coupled to various electrical system busses^72^ and is based on MPMU. To detect UG interruptions, it makes use of electrical parameters. The central controller is made up of logic gate arrays. The outcomes show how selective the system is to non-IS occurrences and how well it can identify IS when there is a power balance. A notable drawback of IDMs based on MPMUs is their vulnerability to assaults originating from communication networks and internet-based data transfers. This problem is addressed in^73^, which promotes the establishment of a specific sub-channel for the sole purpose of detecting islanding. This technique provides defense against many kinds of cyberattacks, such as physical cable blockage, data replication, injecting fake data, and disrupting network traffic.

### Local passive techniques

**Figure 6:**
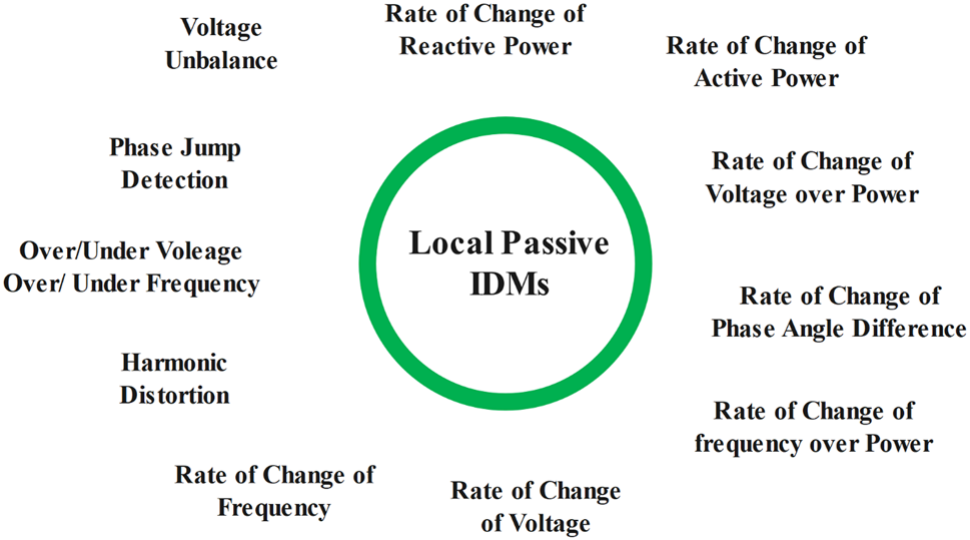
Local Passive IDMs.

A wide variety of IDMs as shown in Fig. 6, that only depend on tracking specific PCC variables are included in Passive IS Detection. Since passive methods don’t cause any disruptions or degrade PQ, its main benefit is that it is non-intrusive. Passive methods have a large number of unreliability issues, nevertheless, mostly because of their large NDZ and sluggish detection performance. OUV or OUF detection, PJD, HD detection, ROCOF, ROCOV, ROCPAD, ROCOP, and ROCORP are some of the important examples that fall under this category^47^.

#### Over/under voltage (O/UV) and frequency (O/UF)

Real-time voltage magnitude and frequency readings are monitored and compared to predetermined thresholds as part of the OUV and OUF schemes. The inverter turns off if it notices an abnormality within a set window of time. With frequency and voltage relays being standard components in most commercial inverters, this approach is among the simplest passive methods.

#### Phase jump detection (PJD)

Since changes in phase dynamics can sometimes happen more quickly than changes in frequency, this method responds more quickly than either the OUV or OUF method^23^. But it's important to remember that improvements in contemporary PLL algorithms have improved the synchronization between the inverter current and PCC voltage, enabling quick absorption of phase jumps and possibly avoiding the detection of islanding. Choosing a suitable threshold to detect grid disconnections presents another implementation challenge. The phase jump threshold is not specified, in contrast to voltage frequency and magnitude^27^. Moreover, the PJD method may result in erroneous tripping since motor starts or capacitor bank switching might generate brief variations in the PCC voltage phase that unintentionally cause detection^74^.

#### Harmonic distortion (HD)

The load impedance, which may be significantly higher than the grid impedance, and the harmonic components of the inverter output current, however, have an impact on the THDv after a grid outage^74^. This method's main benefit is that it is not affected by power imbalances between generated and consumed power^75^. Setting protective relay parameters is a hurdle that comes with its implementation, though. A smaller IS threshold could cause the inverter to trip unintentionally, whereas a bigger threshold could cause the NDZ to grow. It's also critical to recognize that the algorithm is not very selective because some non-IS circumstances have the potential to raise THDv^76^. In addition, the method’s effectiveness is impacted by background distortion in the grid^77^ and non-linear loads^78^. Finally, high-frequency harmonic components may be filtered by local load capacitance, which could lessen the scheme's efficiency. HD-based IDMs have been presented in some research to address the aforementioned issues and improve selectivity, robustness, and efficiency. To increase selectivity in this situation, some techniques combine HD with other passive characteristics. For instance, passive IDMs that integrate harmonic measurement with an unbalanced phase voltage relay are presented in^79–81^. The outcomes show that this combined method successfully distinguishes between IS and non-IS events and delivers accurate IS identification under both balanced and unbalanced load scenarios. Furthermore^82^, suggests a hybrid approach that makes use of THDv and the Gibbs phenomenon. The solution's ability to identify IS development in multi-DG systems is validated by experimental tests. It exhibits a lower harmonic content and a lowered NDZ in comparison to passive HD versions and active IDMs. Other methods identify IS by concentrating on particular harmonic components. A method for HD is presented in^83^ that evaluates the energy density of the third and fifty-fifth harmonic components using a KF, presents an IDM that is based on the inverter’s PWM harmonic signature^84^. Additionally^85^, offers a passive IS detection technique based on the PCC voltage's even harmonic components. The results show how well this approach works in a multi-DG system to identify grid outages. Finally, IS detection is accomplished in^86^ by extracting the second harmonic order of voltage and current using a DFT.

#### Rate of change of frequency (ROCOF)

ROCOF is dependent on several factors, including inertia, nominal frequency, rated power, and the amount of active power supplied by the UG at the moment of islanding. The method can be used using a PLL^87^, ZCD^88^, a Fast FT^87^, an Interpolated DFT and a KF^43^, or PMU^89^. Choosing the right measurement window for ROCOF computation and setting the right threshold for IS detection are two of the main challenges with ROCOF-based IDM. As per reference^90^, the measuring period needs to be between 0.3 and 0.7 s, with a detection threshold of 0.3 Hz/s. Moreover, an approach for constructing ROCOF relays based on field observations made at a biomass production plant is presented in^91^. The intricacy of integrating ROCOF relays with other frequency protection devices presents another obstacle to the successful application of this technique. In response to this problem^25^, suggests a graphical design method to guarantee communication between ROCOF relays and O/UF relays. The susceptibility of ROCOF to annoying journeys after non-IS events is another major disadvantage. As a result, by measuring another electrical variable in addition to ROCOF, various research has tried to address this problem. An under-voltage interlock feature is suggested in^88^ to distinguish between temporary events like voltage dips and grid disruptions. According to^92^, the THDi value is the lock that activates the ROCOF safeguard^93^. Presents the integration of grid impedance estimate with ROCOF. Finally, an improved ROCOF relay utilizing the adaptive KM estimate technique is presented in^94^. This method lessens the susceptibility of the relay to non-IS faults, allowing it to differentiate between grid outages and other electrical emergencies. However, the ROCOF method has a lot of other benefits as well. One such benefit is its adaptability; the scheme may be applied accurately to a wide range of systems, such as PV systems^95^, synchronous generator-based systems^93^, and other kinds of MGs^96^. In addition, some active IDMs that use the ROCOF relay have been created to speed up the detection of grid interruptions: IDM based on RPV implemented with ROCOF relay is introduced in^97^; ^29^ proposes a hybrid technique that combines SFS and ROCOF; and^98^ offers a blend of ROCOF and SMS. Furthermore, in^99^, an active ROCOF relay is suggested.

In contrast to other passive solutions, the ROCOF strategy's NDZ has not been analytically determined in the literature. Nonetheless, several investigations have made an effort to define the NDZ's limits using computational or experimental evaluations. For example, computational research was proposed in^100^ to map the NDZ for PV systems with varied inertial constants and across different RLC loads, spanning different quality factor values. The results demonstrated how the NDZ was dependent on two main variables: the load's quality factor and the amounts of active and reactive electricity that were transferred from the grid to the PCC. On the other hand, in^24^ the blend of ROCOF and ROCOV relay produced a smaller NDZ than the combination of OUF and OUV. Moreover, a comprehensive computational analysis was carried out in^101^ to regulate the upper bound of the ROCOFs NDZ.

#### Rate of change of voltage (ROCOV)

This method depends on the ROCOV, since grid interruptions may cause a momentary divergence in the PCC voltage^22^. Notably, ROCOV can be used for a variety of tasks, such as identifying electrical emergencies or IS occurrences. In HVDC systems^102,103^, and DC MGs^104^, this technique is frequently used for fault detection. Further, as^105^ reports, ROCOV can be applied to AC MGs to detect main loss. Additionally, different IDMs can be combined with ROCOV. One hybrid approach that can identify IS in a multi-DG system is suggested in^106^, which combines ROCOV monitoring with ROCOP. ROCOV relays are considered to be more dependable and selective than conventional current and voltage protection devices, as stated in^107^. Furthermore, a coordinating system based on ROCOV assessment for MGs is proposed in^108^.

#### Rate of change of phase angle difference (ROCPAD)

This method, first presented in^109^, entails tracking the ROCPAD between the voltage and current output of the inverter constantly and examining the rate at which this parameter changes over time. To reduce NDZ, a passive IDM that blends ROCOF, ROCOV, and ROCPAD is proposed in^110^.

#### Other rate of change based-methods

As previously noted, a rapid and brief departure of multiple electrical variables might result from the IS occurrence. Since voltage and frequency are the most frequently occurring, they have their part in this section. Some rate-of-change-based relays, such as ROCOP^111^, ROCORP^112^, ROCOFoP^113^, and ROCOVoP^32^, have been reported in the literature, though. Moreover, a combination of ROCOP and ROCORP is suggested in^114^.

#### Voltage unbalance

A three-phase system may encounter phase delays or voltage amplitudes that deviate from 120 degrees, which can lead to a phenomenon known as voltage unbalance. The loads in each phase of an IS operation may also have varying magnitudes or impedances, which might result in voltage imbalance. For instance, as was previously indicated, in^79^, the voltage imbalance is utilized to interlock the IS analysis with the harmonic measurement. A comparable method was suggested in^80^. To enhance the ability to discriminate between IS and non-IS occurrences, it measures the voltage unbalance with the fifteenth order harmonic. Furthermore, a few hybrid techniques operated by using the voltage imbalance as a passive stage. These methods will be further examined in the subsection on hybrid methods.

### Local active techniques

As previously stated, when there is a balance between the power produced by DG and the power required by local loads, passive IDM shows unreliability. Consequently, active IDMs were developed in response to the requirement to overcome NDZ difficulties with passive IDMs. To alter the operating point outside of the thresholds of the established standard, these strategies entail producing perturbations in the inverter operation. Although active solutions inevitably lead to a decline in PQ, their use is warranted by the reduction of NDZ^28^.

#### Active frequency drift (AFD)

The main benefit of the AFD solution is easy to implement. It's crucial to remember that this algorithm has several shortcomings, such as high levels of THDi, inefficiency in multi-DG IS circumstances, serious NDZ problems and impact on PQ due to frequency perturbation. An additional constraint related to the fixed value of C_*f*_ is present. As a result, there is an issue with the load-induced frequency wandering propensity. More inductive loads tend to cause the frequency to drift below the grid frequency, whereas when C_*f*_ is positive, the frequency tends to drift towards values higher than the grid frequency. The IDM's imposed frequency deviation may be countered by this load-induced frequency drifting tendency, which could lead to a failure to identify grid outages.

#### Improved active frequency drift (IAFD)

The IAFD algorithm was proposed by^115^ in order to address the THDi issue with the Classic AFD. During the odds quarter-cycles of current, IAFD substitutes a step on the current magnitude for the dead time. From an analytical standpoint, it can be inferred that the algorithm's performance in terms of incursion can be dictated by the value of the parameter K. The detecting capability will increase with increasing K, but the output current's harmonic content will also increase with increasing K which will impact on to PQ.

#### AFD With pulsating chopping factor (AFDPCF)

As stated before, the Classic AFD is unable to adjust to the frequency drifting brought on by local loads. In order to overcome this restriction^116^, created the AFDPCF, which substitutes a pulsing signal varying between positive, zero, and negative values for the fixed value of C_*f*_. This method’s main advantage is that it reduces the THDi injected during the conduction periods when C_*f*_ equals zero. As demonstrated in^117^, the AFDPCF algorithm ultimately eliminates the NDZ for a variety of values. The AFDPCF design process, which computes C_*f max*_ and C_*f min*_ values to eliminate a certain range of quality factor values, was also introduced by^117^. The algorithm's main drawback, though, is that it takes longer to detect ID than other approaches. As explained in^117^, the change in the C_*f*_ value and the grid disruption do not occur at the same time. This means that the approach can only start frequency drift after the C_*f*_ value changes, if IS occurs when C_*f*_ equals zero^118^. Discusses an alternate version of the AFDPCF.

#### Sandia frequency shift (SFS)

The SFS method was put forth by^119^ to fix the Classic AFD algorithm's operational issues by using a variable chopping factor that is connected to the detected frequency inaccuracy. An inverter output current phase discrepancy concerning the PCC voltage is produced by the distortion introduced into the inverter output current. THDi rates and T_D_ are reduced, which is the main benefit of the SFS method. In contrast, the frequency differs from the nominal value during a UG outage, which causes C_*f*_ to rise. This frequency variance intensifies C_*f*_ even more, resulting in a feedback loop that shortens the T_D_. It is evident that C_*fo*_ and the accelerating gain K are the two parameters that determine the SFS design. The gain K establishes the NDZ size, whilst the C_*fo*_ influences the THDi rate^120^. Therefore, the application of SFS aims to achieve the greatest NDZ reduction with the least amount of THDi injected. Numerous design techniques have been suggested for this situation. But the amount of inverters linked to the same PAC can affect the SFS algorithm’s NDZ, which reduces the algorithm's capacity for detection. To solve this issue and ensure the best possible tuning of the SFS even in multi-DG cases^121^, suggested a new design technique for the SFS scheme. Although computational models were successfully completed, no experimental validation was given.

A dynamic analysis was conducted on the NDZ problem by^122^ to investigate the impact of the accelerating gain K and the parameters C_*fo*_ on the NDZ mapping and size. The range of Q_*f*_ values for which the NDZ is eliminated was found to be directly impacted by K; so, an increase in K results in a smaller NDZ. Conversely, C_*fo*_ establishes the Cnorm's value at the NDZ's starting point. In this way, a rise in C_*fo*_ corresponds to an increase in the norm. However, the stability analysis carried out in^13^ proven that high K values can have an impact on the converter's stability and cause incorrect tripping, especially for large-scale DGs or weak grids. In this case^123^, suggested an SFS variant that is comparable to the AFDPCF algorithm and is based on the idea of the pulsing C_*fo*_.

A different method of parametrization involves selecting C_*fo*_ and K correctly by applying ML and artificial intelligence techniques. An adaptive FL-based approach, for example, estimates the local load parameters in^124^ and uses the estimated parameters to find the lowest value of K which eliminates the NDZ. This process follows SFS parametrization guidelines. An ML method based on the immune system, however, makes the parametrization in^125^. THDi and T_D_ in a PV and wind power DG system were better in the experimental realization.

#### Slip mode shift (SMS)

By inserting a tiny disturbance through a frequency positive feedback loop into the phase predicted by the PLL, the Slip Mode Frequency Shift is achieved. The primary benefits of this IDM are its capacity to follow the frequency variation of the load applied on it, its digital implementation's ease, and the removal of the NDZ for a certain factor of Q_*f*_^98^. A comparative analysis of how multi-DG IS affects the SMS strategy's NDZ is suggested in reference^6^. The study discovered that multi-DG operation increases its NDZ and negatively affects performance. The novel APS technique is proposed in^126^ and introduces an initial fixed perturbation. A modified APS is suggested in^127^, where the parameter values change based on the local load's estimated impedance. A method of reducing the T_D_ involves combining the SMS with the ROCOF algorithm, as suggested in^128^. The hybrid IDM presented in^129^ is based on combining the SMS with the Q-f droop curve; the next subsection will go into great depth about this method. In an experimental comparative analysis^130^, shows that, in comparison to the SFS scheme, the SMS achieved faster IS detection.

#### Sandia voltage shift (SVS)

The SVS algorithm is designed to identify IS by introducing an interference into the inverter's current reference, which causes a voltage drift in the PCC voltage following a grid outage^47^. The ability to identify IS even when there is a discrepancy between the power required by the load and that provided by the SGD is the primary benefit. Thus, it can be concluded that the approach experiences less interference from the reactive load characteristics once the voltage is less affected by the reactive power flow of the grid. Conversely, the primary drawbacks of the SVS are its effects on inverter stability and decrease PQ^131^. A MSVS that employs an exponential change in the gain of the voltage positive feedback was presented by^132^ in an attempt to lessen the method's shortcomings. Furthermore, the technique can be used in tandem with the SFS to enhance the capacity to diagnose IS^133^. This approach was compared to other well-known IDM in^130^, and it produced a proper IS detection with only a minor PQ disturbance. This comparison analysis did find, however, that the SVS algorithm is slower than other IDM alternatives like AFD, SMS, and SFS.

#### Reactive power veriation (RPV)

A novel IDM is put forth in^134^ that involves tampering with the reactive power reference to produce an imbalance in reactive power between the local load and the inverter. Using a Q-f droop curve, an IDM is suggested in^135^ that operates by positioning the inverter operation in an unstable operational state after IS occurs. Active techniques to differ the frequency outside of the permitted range are proposed in^136,137^, whereby the q-axis current control is perturbed^136^. Employs an intelligent control to ensure quicker ID^137^, put forth a method based on the dq reference frame perturbation that may ensure selectivity and prevent the inverter from tripping for non-IS contingencies even while operating under weak grid conditions. A hybrid IDM with four passive criteria is presented in^138^ to help choose when to inject the RPV disturbance. A novel hybrid IDM is given in^81^ that combines an active bilateral RPV with two passive features, namely voltage imbalance and THDi. This approach represents a significant advancement over^138^, as it accurately detects IS while requiring less computing power due to its two-variable measurement requirement. Finally, an altered Q-f droop curve-based IDM is put out in^139^. The results of the experimental validation demonstrated that it can eliminate the NDZ for a variety of Q_*f*_ values, prevent false inverter tripping, and exhibit strong performance for multi-DG islanding. A bidirectional intermittent RPV-based IDM is proposed in^140^. A new RBV-based algorithm is proposed in^141^, and its parametrization is related to the frequency resonance of the load. In a multi-DG system, the techniques of^140,141^ might not be as successful in detecting islanding, despite their good performance. Furthermore, non-unitary power factor inverters are incompatible with any approach. A unique RPV-based IDM with the addition of two sets of RPVs was presented by^142^ in this particular scenario. The outcomes demonstrate that this approach can identify grid outages for power factor inverters with unity and those without, in both single and multi-DG systems.

#### Harmonic injection

The interplay between the inverter current and the impedance of local loads causes the associated voltage harmonic order to grow after a grid outage^47^. This method’s principal benefit is its independence from the balance between electricity consumed and generated^75^. Nevertheless, choosing a safe threshold to identify IS is one of the primary disadvantages. Furthermore, for loads with high filtering capabilities, this approach may encounter NDZ. In addition, backdrop distortion may cause annoying excursions, instrumentation noise, or other non-IS events.

A new PLL method based on the SOGI was presented in the publication^143^ for this particular circumstance. The same authors suggested an IDM in^26^ that relies on injecting a harmonic signal that can be roughly represented by a double-frequency oscillation. Papers findings shows that even for an RLC parallel load with Q_*f*_ < 10, the technique may detect islanding. IDM inserting a very comparable disturbance is proposed in^11^. However, GA lowers the number operation and, as a result, provides the measurement of the second harmonic order disturbance. Although the GA is utilized in^144^ as well, the suggested approach is predicated on the addition of the ninth harmonic component. The experimental findings demonstrated a strong performance in IS detection. However, it should be noted that the strategy is more susceptible to inverter false tripping than other approaches that use lower harmonic orders because the threshold for the 9th harmonic amplitude is only 2%^14^.

In order to assess the grid impedance, an IDM specifically designed for three-phase inverters is proposed in^145^. It involves inserting two non-characteristic current harmonics. A digital processing algorithm is used in this strategy to address the issue of instrumentation noise-induced nuisance trips. Additionally, it can identify IS even when there is a power balance. To prevent disruption filtered by capacitive loads, the authors of^146^ employ a subharmonic injection. To reduce the T_D_, an IDM system based on the feedback of the voltage harmonic's rate of change is put forth in^147^. Additionally, it makes use of a binary tree classification technique to prevent the inverter from false tripping.

The study^10^ examined the issue of HI-based techniques in a multi-DG system in depth, taking into account both the DGs actual configuration and potential for extension. Ultimately, it also offers an external integrator-based method that optimizes the UF and raises the HI IDM solutions' dependability. In^148,149^, the compatibility problems of HI techniques in multi-DG systems are also examined. As per the findings of^148^, the suitability of HI methods in a multi-DG setting is dependent on the phase difference between the disturbances introduced by every inverter unit lying within the range of [− π/2; π/2]. This study presents a novel solution to this problem, which is to introduce high-frequency current components of negative sequences for single and three-phase inverters. Consequently, this approach is adjusted in^149^ so that it can be used on DGs with grid-connected transformers.

The difficulty of obtaining precise harmonic information is another issue with the HI process. Strategies that require intricate mathematical processes, like the aforementioned GA, DFT, or ML approaches, are typically used. However, other authors proposed cross-correlation-based strategies to reduce the computational complexity. For example, an IDM is proposed in^150^ that uses the correlation factor between the inserted current disturbance and the effect it has on PCC voltage to detect grid interruptions.

Although this technique works well, it does not analyze the effects of the grid characteristics and may cause flicker issues or interfere with the DC voltage management. Lastly, a new IDM is proposed in^151^ that use a cross-correlation technique to extract the signal and adds a second-harmonic current component. Because the correlation examines features of the natural grid, it ignores the need to monitor the injected current. Furthermore, even with pseudo link DC, this technique is appropriate for Module Integrated Converters (MIC) and has a modest NDZ.

#### Negative-sequence current injection

This method creates a voltage imbalance between the system's phases following an IS event by injecting a negative-sequence current into the inverter output. Since this method can only be used in three-phase DGs, its principal disadvantages pertain to portability. Furthermore, it causes issues in systems with unbalanced loads for the same reasons as well as in multi-DG IS because of the potential mutual cancelation of the introduced disturbance. Additionally, it was determined in^152^ that the scheme is vulnerable to nuisance trips due to load fluctuations, rotating machine switches, and other non-IS occurrences. An IDM is presented in^152^ that measures the negative-sequence voltage at the PCC to diagnose islanding. The provided results pertain solely to computational simulation and demonstrate the ability to identify IS within 60 ms. The approach is proven to be insensitive to variations in load characteristics by taking into account the IDM testing criteria. The NDZ of the suggested solution in^152^ is found in^153^. Furthermore, a modified approach that can exclude non-detectable situations is proposed in the research, raising the disturbance magnitude to 5%. Current injection method in a multi-DG setting that can identify islanding. An approach for sequential negative current injection that can identify IS in a multi-DG setting is presented in^154^. A new negative-sequence-based IDM with a positive-feedback disturbance is proposed in^155^. The collected findings show that the method does not depend on Q_*f*_ influence, operates in varied grid situations without the need for parametrization modifications, and decreases PQ degradation when compared to previous negative sequence-based IDM approaches. Conversely, the largest disadvantages are the low T_D_ and the heavy computing load. Lastly^156,157^, offer additional methods based on negative sequence injections. However, a high penetration rate of PV inverters can negatively impact their performance, and in weak grid situations, they are susceptible to trips from annoyances.

#### Modern positive feedback methods

A novel active IDM known as APJPF is presented in^120^. It links the inserted distortion with the frequency error^45^, by combining the distortion suggested in^158^ with a frequency positive feedback. On the other hand, a comparison of the solution suggested in^120^ and other schemes is conducted in^117^. Additionally, the study suggests a parametrization approach to determine the lowest K gain required to ensure the NDZ is eliminated for a particular range of Q_*f*_. According to the experimental findings^117^, outperforms AFDPCF, AFD, AFD by^158^, and SFS algorithms for a single DG environment in terms of THDi, T_D_, and NDZ. However, this IDM technique still lacks a multi-DG analysis.

A novel approach to IS detection utilizing phase-shifted feed-forward voltage is presented in^159^. Its working concept is comparable to that of the SMS algorithm. The experimental findings confirmed the IDM's effectiveness for several quality criteria in both scenarios. A frequency positive feedback technique that introduces a disturbance on the control reactive power reference^160^ is based on the droop curve. The major objective is to alter the frequency beyond the permitted operational value range to ensure proper inverter shutdown following the occurrence of islanding. The plan passed every test under 90 distinct load situations (varying power levels, normalized capacitance values, and quality factor values), and it achieved accurate identification in every scenario. Nevertheless, more research is required to evaluate the method's effectiveness in multi-DG systems.

### Local hybrid techniques

Compiling the average benefits and drawbacks of both passive and active techniques, the local hybrid IDM. In this way, when compared to active schemes, they can lessen PQ degradation, and when compared to passive schemes, they can lessen NDZ. The principal shortcomings, therefore, are the lengthening T_D_ and the rise in complexity. Their method of operation is split into two phases. The passive approach is the first when an electrical variable is the only thing being monitored. To verify an IS occurrence, the active stage is initiated if the IDM detects one. If not, the active phase is turned off. The primary hybrid IDMs' timeline evolution is discussed in this subsection.

#### SFS based hybrid methods

A hybrid method in^29^, wherein the ROCOF monitoring causes the SFS disruption. Since ROCOF's dynamics are faster than those of frequency, it can help improve the T_D_, which is one of the key shortcomings of hybrid systems. The results show that for loads with Q_*f*_ ≤ 5, the technique can detect islanding. Furthermore, the approach demonstrated strong performance in multi-DG contexts and weak grid situations, with the ability to differentiate between IS and non-IS eventualities. A hybrid approach is presented in^161^, wherein reactive power monitoring triggers the SFS disturbance. Furthermore, it makes use of APSO to determine the ideal gain K value to minimize stability implications and ensure accurate IS identification with the lowest possible THDi. Computing outcomes suggest that IS may be identified under power mismatch load circumstances and Q_*f*_ = 2.5. Nonetheless, further testing is required to validate the algorithm’s capabilities.

#### SMS based hybrid methods

A novel hybrid IDM is proposed in^162^, wherein the active stage introduces the same phase disturbance as the SMS algorithm. The frequency estimation that a droop control performs, in turn, activates the passive stage. Using a computer simulation, the suggested algorithm was evaluated under the UL1741 Standard and was successful in correctly detecting IS for both single- and multi-DG systems at various quality factor levels. A comparable method capable of performing IS diagnostics in a multi-DG context is proposed in^163^.

#### Voltage unbalance methods

Voltage imbalance can result from the IS phenomenon in a three-phase system, as was previously mentioned. Voltage imbalance is employed by certain writers as the passive phase in various IDMs. Reference^30^ suggests an IDM that combines frequency perturbation and the idea of voltage unbalance monitoring. Three passive variations are monitored passively in^138^: voltage imbalance, ROCOF, and voltage variation. The active step of this hybrid technique is based on the disturbance of the inverter's reactive power. When the frequency variation and the introduced perturbation no longer go below a predetermined threshold, IS is recognized as having occurred. With no requirement for inter-inverter communication, the computational findings show efficacy for a range of load scenarios and IS detection capabilities in a multi-DG context. An IDM is proposed in^81^ that combines active bidirectional RPV with passive voltage imbalance and active THD detection techniques. It also offers a precise process for selecting the harmonic detection threshold appropriately using circuit analysis. The outcomes demonstrate excellent performance in multi-DG situations and compliance with the^2,164^ criteria.

#### ROCOF based methods

The passive stage of hybrid IDM can be effectively implemented using ROCOF monitoring, which is a potent tool for passive IS identification. Apart from the previously discussed references^29,165^, wherein the ROCOF functions concurrently with the SFS and SMS, respectively^166^, suggests a hybrid approach consisting of passive ROCOF monitoring and active manipulation of the inverter's reactive power output. Results show that this method detects IS more quickly than previous methods mentioned in^99,141^.

To detect IS in a DG system, a novel hybrid IDM based on ROCOF is proposed in^165^. The suggested approach integrates the elements of the ROCOF passive monitoring system with the perturbation suggested in reference^99^. Utilizing the MATLAB/Simulink environment, the suggested method was implemented. Tests are still required to confirm the strategy even though the simulation results show that the method can identify imbalanced IS in less than 100 ms and zero-power mismatch IS in 200 ms.

#### Other ROC based methods

As was already established, rather than monitoring the observed electrical amount itself, passive IDM watch its derivative. In this case, achieving the passive stage of hybrid approaches can also be done by measuring the rate at which an electrical variable changes. A hybrid IDM using the ROCOV as the passive stage is proposed in this context by^32^. To diverge the voltage magnitude beyond the permitted range of operation, a RPS is started when the detected ROCOV value rises above a particular threshold. In this regard^106^, suggests a hybrid approach that makes use of the ROCOV as the passive stage and the inverter output active power perturbation as the active one. The technique was evaluated in a variety of scenarios, including the energization of transformers, the starting of induction motors, and the functioning of several DGs. The findings show that the approach can identify IS for loads falling inside the quality factor range of 0 < Q_*f*_ ≤ 5 and is specific for non-IS events. Nonetheless, further research and development are required to validate the approach.

### Intelligent passive techniques

**Figure 7:**
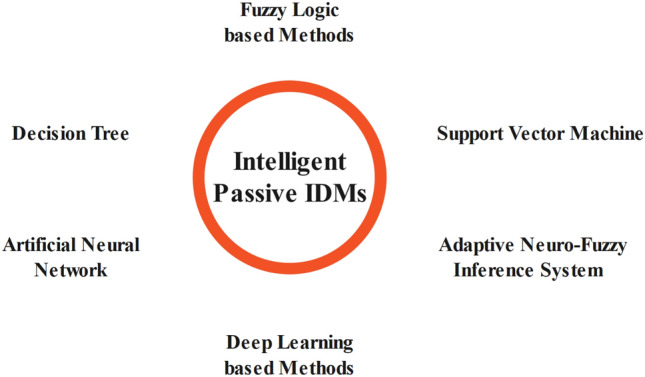
Intelligent Passive IDMs.

As was previously said, selecting the right threshold for detection is a challenge for many techniques used to identify power system events. While standards specify recommended thresholds for frequency- and voltage-based schemes, the application of other approaches, such as ROCOF, ROCOV, HD, and HI, is characterized by the challenge of choosing the appropriate threshold for detection. Under this situation^46^, predicts that IDM research would probably move toward using intelligent classifiers as shown in Fig. 7, including SVM, ANN, FL, DT, and ANFIS. Because they extract features from the signal and use them as input for judgment, these classifiers do away with the difficulty of setting detection thresholds. It is noteworthy that certain active approaches, such as those covered in^124,161^, use ML and its algorithms. Though they don't directly affect the IS decision, they are left out of this part because the main goal of using ML is to dynamically parameterize the techniques.

#### Decision trees

DTs are a subset of ML algorithms that use feature-based binary classification to evaluate data inputs and provide conclusions. DT can be taught to identify the electrical signals connected to IS by providing instances of both regular grid functioning and IS occurrences. The DT can be taught to identify fresh data and, if an IS event is recognized, can be utilized to isolate DG, therefore enhancing grid safety and dependability^167^. A DT-based method is presented in^168^ that avoids falsely tripping the DGs by processing the signals using WT and extracting characteristics from transient variations in PCC voltage and inverter output current. When it comes to IS detection, the acquired findings outperformed passive options like the ROCOF relay and the OUV/OUF approach. It is important to note, nevertheless, that whereas the ROCOF method and OUV/OUF attained a 100% selectivity rate, the DT solution only managed a 93.75% selectivity rate, with an average T_D_ of about two conduction cycles. The DT + WT combination is also used in the technique suggested in^169^ to carry out loss of mains detection. An approach based on the Random Forest Classification (RFC) concept is put forth in^35^. Alternatively, by employing just four conditions, the computational overhead can be decreased. However, the overall efficiency drops to 98%. A DT-based IDM is presented in^170^, where the chosen features are placed in several measurement windows based on their specificities. This is a significant benefit in terms of T_D_, as it enables the quick classification of incidental IS scenarios, when an imbalance between energy generation and consumption is not confirmed. The findings show an accuracy rate of > 99%, and 79% of the evaluated cases had T_D_ that were less than 20 ms.

#### Artificial-neural-network

**Figure 8:**
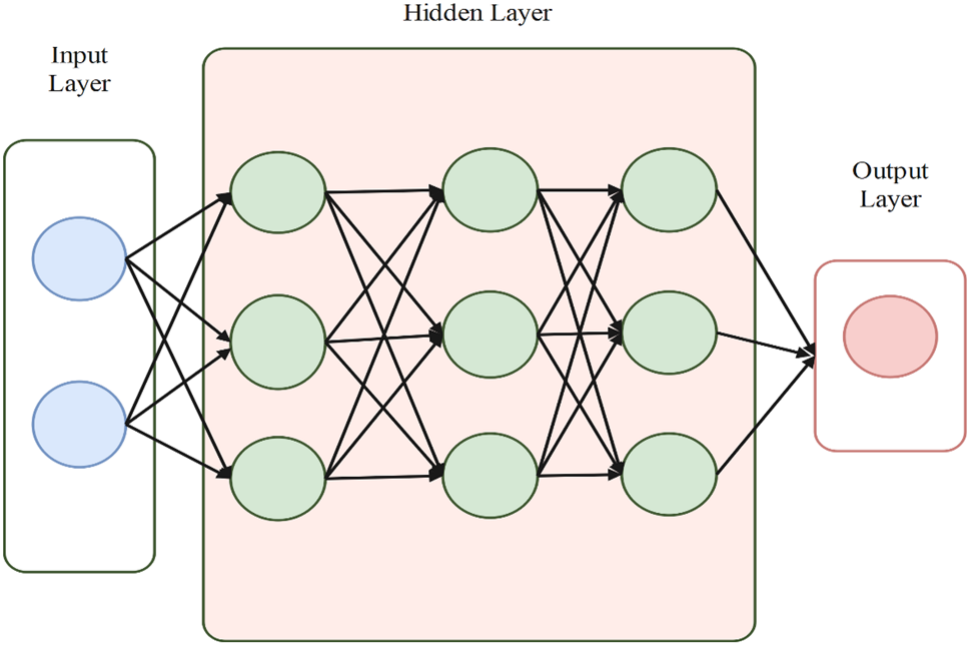
Generic ANN representation.

An ML technique known as an ANN is composed of many interconnected processing units, or neurons, arranged in layers. Figure 8 illustrates how each neuron gets input signals from other neurons and generates an output signal that can be utilized as input to other neurons in subsequent layers^171^. Feature extraction is a crucial phase in the training of ANNs. Phase space, DWT, HT, TQWT, and DFT are the primary techniques used to extract features^48^. It is suggested in^172^ to use an ANN-based IDM that uses the GreyWolf Optimized ANN as the intelligent IS classifier and the VDM and HT to facilitate feature extraction. With two synchronous machines and two PV systems, the system was tested in a multi-DG system scenario. The calculation time, robustness against noise conditions, and accuracy of IS classification are all effective, according to the results. A two-stage mechanism for detecting IS is proposed in^41^. The first step involves using the DFT to extract features from recorded voltage and current waveforms. The second stage involves classifying the existence of IS using a KNN-based classifier that receives nine features as input.

The classic method for a single-hidden layer FNN, is recommended to be replaced by a new ANN-based IDM in^173^. Analytically, it determines the output weights by selecting the input weights and hidden layer biases at random. More accuracy was obtained for both IS and non-IS occurrences when the suggested method's findings were compared with the DT-based IDM. To achieve a total accuracy of 99.09%, the ELM approach is also used in^174^. Certain methods employ the WT to ensure accurate feature extraction. An IDM that employs both WT and MM in the extraction stage is presented in^175^. An ELM is in charge of the classification. Depending on the size of the training data set, the produced result, which has an accuracy of 100%, demonstrates the usefulness of the proposed IDM even in very noisy environments. In the paper^176^, an IDM based on ANN is presented. TQWT is used to extract features. The WT method known as TQWT allows for the adjustment of a control signal's oscillatory behaviors through configurable control parameters. The algorithm's parameterization process is also presented in the publication. 98% efficiency for both IS and non-IS occurrences is shown by computational studies.

#### Suport vector machine

Regression analysis and classification are two applications for supervised learning techniques called SVM. SVM seeks to identify the hyperplane that divides the data into classes as much as possible, leaving a margin between the nearest data points in each class. Because SVMs are capable of processing both linear and non-linear data, they are frequently used in electrical power system protection, particularly for event categorization and IS detection^177^. When compared to the previous ANN performance, this method offers a significant advantage. The way it works is based on the idea of Structural Risk elimination, which goes beyond eliminating training data error to instead focus on minimizing an upper bound on the projected risk. As a result, using a smaller training sample can lead to improved accuracy^37^. Within the framework of IDM, several SVM-based methods might be emphasized. The following features are extracted using the IDM presented in^178^: voltage, frequency, voltage phase angle, ROCOF, and ROOV. When the active power imbalance is 5% or more, the method effectively identifies IS events; however, when the imbalance is less than 8.8%, the VS relay malfunctions. An SVM-based event classification approach that can be applied to the identification of IS is proposed in^179^. Under UL741 testing conditions, the IDM demonstrated efficacy, selectivity, accuracy, and precision. The solution outperformed results obtained by ANN-based algorithms, with an average T_D_ of 40 ms.

#### Fuzzy logic

FL can be used for IS identification in the context of electrical power networks^180^. A FL-based IDM that performs IS detection in two stages is proposed in^181^. To diagnose loss of sources, the first one is made up of a DT algorithm that takes features and selects the three most important ones. IS and non-IS electrical events are divided into two classes by the FL classifier, which makes up the second stage.

#### Adaptive neuro-fuzzy inference system (ANFIS)

Combining the best features of ANN and FL classifiers, ANFIS algorithms are a hybrid technique. ANFIS is a versatile tool that may be utilized for a range of tasks, such as electrical fault classification and IS detection. These jobs include regression, control, and classification^182^. An ANFIS IDM specifically for wind turbines is presented in^183^. Selectivity to non-IS events and accurate diagnosis of source loss in 1.5 s is demonstrated by the experimental validation. Conversely, Paper^184^ presents a novel technique for passive IS detection through data clustering, wherein subtractive clustering is used to build a robust and simplified fuzzy classifier. The ROCOF relay was outperformed, according to the results. A method for ANFIS extraction of seven PCC inputs is presented in^38^. Testing of the procedure was conducted with UL741 standards. Findings show a 78.4% success rate for accurate IS detection. Furthermore, it exhibits a quicker detection than the other tactics in this section. While selectivity and efficiency are indicated by the data.

#### Deep learning based methods

DL is an ML method that learns representations of data with various degrees of abstraction by utilizing numerous processing layers. The idea of DL, based on a two-step process for training deep structures utilizing unsupervised pre-training and supervised fine-tuning, was put up as a solution to the issue^185^. A proposal for using DL for IS detection is found in^186^. The suggested classifies whether or not an IS event is present using a regression method and a deep neural network built on stacked autoencoders. The findings show a 98.3% accuracy rate and a 0.18-s T_D_. Compared to alternative classification techniques, this method performs better but requires a very high sample size. A two-stage DL technique is proposed in Reference^86^. In the first, a DFT is applied to voltage data detected from the PCC to ensure selectivity and extract features specifically linked to IS events. The ability to identify IS in 6 ms with an average accuracy rate of 99.61% was shown via computational testing. The outcomes also show that this strategy is better than others, including DT, SVM, and ANN, in terms of accuracy and T_D_. This paper^34^ proposes a new DL-based IDM that consists of four stages: obtaining voltage data in three phases, concatenating the data, converting the data into time variations in the frequency spectrum using the short-term FT, and feeding the phase and magnitude data into a DNN to classify IS and non-IS events. The outcomes show a 98.76% efficiency rate. Furthermore, it could accurately diagnose IS in a multi-DGs context without compromising PQ. Ultimately, selectivity tests were conducted on it to demonstrate its ability to differentiate between non-IS contingencies.

### Signal processing based methods

**Figure 9:**
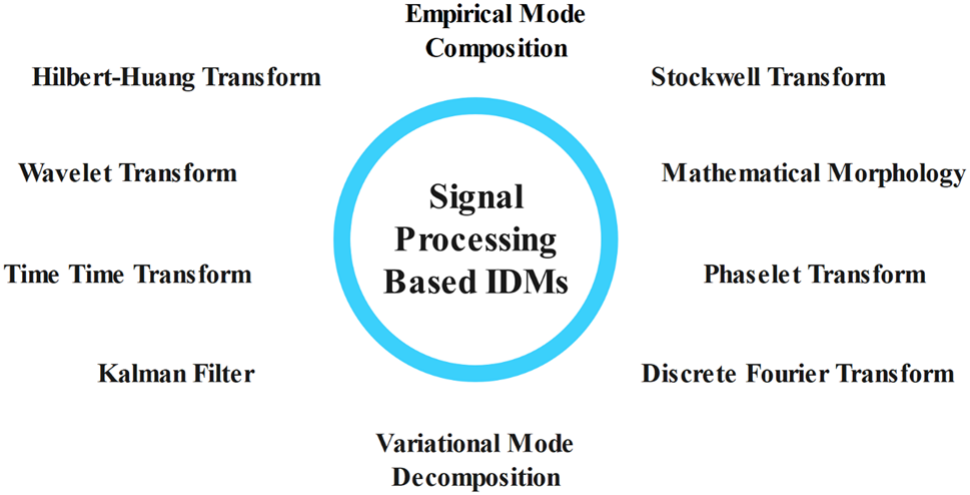
Signal Processing based IDMs.

SP methods as shown in Fig. 9 can be applied to improve passive methods of IS detection. These techniques offer cost, stability, adaptability, and flexibility, which help researchers identify hidden characteristics in recorded signals for IS detection. Based on these detected qualities, decisions can be made on the likelihood of islanding. To detect islanding, several SP tools are used, including the FT, ST, HHT, WT, and TT-transform; they are explained in more detail in the sections that follow^49^. It is important to note that, as was previously said, some intelligent algorithms use SP technologies to extract features. This subsection will not address these techniques.

#### Wavelet transform (WT)

The signal is represented in both the temporal and frequency domains as a result of this decomposition, making it possible to identify localized features. The WT is an adaptable tool that may be used to analyze signals of any size and detect changes over short and extended periods. Its uses go beyond SP and include data extraction, audio analysis, and image processing. WT has been used in the field of electrical power systems for fault categorization and IS identification. The first use of WT for IS detection has been reported in^187^. This technique uses WT to separate high-frequency harmonic components by monitoring the inverter output current and the PCC voltage. Results from computation and experimentation show that the approach can detect source loss in less than 400 ms, especially when the grid's active and reactive power contributions are both zero. A new approach to Active IDM based on WT is presented in^188^. It uses the advantages of WT, SVD, and Shannon entropy to assess transient changes in PCC characteristics. Detailed coefficients are obtained by applying WT to three-phase voltage signals. These coefficients are then utilized to build a singular value matrix and calculate the WSE for every phase. The WSE index is then calculated, which is the total of the WSE values from every phase. The proposed method has been found to provide increased selectivity and faster detection of IS occurrences when compared to traditional ROCOF and ROCOV relays.

WPT is used in^189^ to extract features from the apparent power of the three-phase system to implement an Active IDM. A combination of wavelets called WPT is useful for capturing high-frequency harmonic component transient oscillations. In a hybrid DG configuration with a grid-side converter, a permanent magnet generator, and a wind turbine simulator, the IDM was assessed. The results of the experiments show an average T_D_ of 10 ms, as well as acceptable low-voltage ride-through performance and minimal effect on PCC PQ. Furthermore, an analogous IDM is used in a cogeneration plant by Paper^190^, and a similar technique is applied in farm collector systems by^191^. Based on WT the Active IDM is presented in^192^. It changes the traditional continuous WT. IS pattern detection entails the examination of a dataset that contains multiple PQ variables. A wind power plant, a battery bank, a photovoltaic system, and a synchronous generator made comprised the MG setup used to evaluate the method. The results show that it can differentiate between various electrical occurrences including two-phase failures, phase-to-line short circuits, and IS events. Another novel approach to Active IDM is presented in^42^, which involves modifying the discrete WT. This technique uses a Maximum Overlap Discrete Wavelet Transform (MODWT) and a Second-Generation Wavelet Transform (SGWT) in place of the conventional discrete WT. Reduced memory usage, less computational load, and fewer pointless calculations are the goals of the SGWT. In the meantime, the length of the signal is maintained because the MODWT stops the sample size from decreasing at every stage of decomposition.

#### Mathematical morphology

The time-domain analytical technique known as MM focuses on signal form, integral geometry, and set theory. When compared to other SP techniques like WT, ST, HST, and TTT, morphological filters which are based on MM have lower computational needs since they use simple signal transformation operations like addition and subtraction. Because of its simple implementation, it can also be used for fault categorization and source loss detection in DG^39^. The MM operator dilation and erosion are used in^193^ to produce a DED filter, which is in charge of extracting IS patterns from the PCC voltage and the DGs output current. This is a proposed MM technique of IS detection. To identify islanding, the MMRI, which is derived from the DED filter's output, is compared to a predetermined threshold. An average T_D_ of 20 ms is shown by computational results. A comparable method that develops an IDM based on MM specifically for MGs is suggested in^194^. It detects IS solely by measuring the PCC voltage, although employing the same DED filter idea. The results of the computation show that IS detection is possible in less than 10 ms on average.

#### Stockwel transform (ST)

The WT shortcomings such as batch data processing and noise sensitivity are suggested to be remedied by the ST. Short-time FT and WT are combined, using a scalable Gaussian variable window. With the help of this technique, a time series signal can be converted into a time–frequency representation with frequency differentiation. Multi-resolution is possible with this method without changing the phase of the different frequency components. ST makes use of the amplitude or phase time–frequency spectrum to help identify disturbances like electrical faults or islanding, as well as to observe local spectral patterns^51^. The ST is used in^40^ to assess the inverter output current and produce a MIRF, which is the first step in the Active IDM. To determine the CIRFC, the RMS current is differentiated over time to determine the rate of change of MIRF. To separate IS events from defective and operational events, simple decision rules are applied to compare the peak magnitude of CIRFC with established threshold values. For IS contingencies, two thresholds are set: one for fault detection. Assuming a signal-to-noise ratio of 10 dB, the results show selectivity for non-IS events and precise identification of IS. In^33^, a technique is put forth to extract negative sequence data from voltage and current signals and construct an indicator for current IS detection by combining the ST with the HT. This method, which was tested with an IEEE-13 nodes model, achieves an efficiency of more than 98% and can successfully identify IS events even in noisy surroundings with a signal-to-noise ratio of 20 dB. A comparative analysis was also carried out, which shows that the suggested approach outperforms WT-based algorithms, traditional passive solutions, and ANN-based IDMs. A technique for identifying events as IS or non-IS is introduced in^195^. It makes use of ST-based multi-resolution analysis to evaluate voltage signals and determine the variation index and standard deviation. The system performs well in noisy situations and can distinguish between non-IS events and grid interruptions. Software from MATLAB/Simulink was used to conduct the investigation, and a real-time digital simulator was used to validate the results in real-time. The findings show that IS occurrences and temporary, normal changes in electrical amounts can be distinguished with accuracy.

#### Empirical mode composition (EMC)

An adaptive multi-resolution SP method called EMC can be used to separate non-stationary or non-linear signals into distinct groups of IMFs at different resolutions. In^45^, IS detection was achieved by the use of EMC. A novel approach to Active IDM is presented in this work. It is based on a Time-Varying Filter (TVFEMC), which uses an adjustable cutoff frequency filter to create two IMFs. A Teager energy operator is used to calculate the IMF's energy density; the output is then compared to a preset threshold to identify islanding. Results show the capacity to identify IS events at 40 dB signal-to-noise ratio with selectivity for non-IS events.

#### Hilbert–Huang transform (HHT)

A SP technique used to analyze non-stationary and non-linear time series data is the HHT. Hilbert Spectral Analysis (HSA) and EMD are the two processes that make it up. An EMD breaks down a signal into a collection of IMFs, or intrinsic mode functions, that describe different frequency components in the signal. The HT is employed by HSA, in contrast, to investigate the instantaneous frequencies and amplitudes of these IMFs. Because HHT can analyze non-stationary signals, it can capture changes in frequency content and amplitude over time, which makes it useful for IS identification^51^. In^196^, an HHT-based Active IDM is unveiled. It entails monitoring the inverter output current and PCC voltage and then analyzing these signals using EMD to obtain IMFs. After computing the HT for both voltage and current signals, a ratio index is obtained from these transforms. The ratio index functions as a cutoff point to detect IS events and differentiate them from switching events that are not islanding. Even with a 5% discrepancy in reactive power, the suggested method was able to identify islanding.

#### Variational mode decomposition (VMD)

A method for SP called VMD breaks down the input signal *u*(*t*) into discrete signals *u*_*k*_(*t*), also known as band-limited IMFs or sub-signals. When compared to other methods, it provides a more accurate and stable signal breakdown, making it possible to distinguish and isolate signal components with different frequency content and amplitude modulations. VMD has proven to be effective in a variety of sectors and can provide novel perspectives on problems in science and engineering. VMD algorithms can be used in the context of DG and MGs for fault classification and IS detection. Five IMFs are extracted from the PCC voltage by the novel Active IDM described in^197^. This algorithm makes use of the VMD idea. Statistical parameters like standard deviation and Kurtosis index are then calculated by a control system. Comparing these characteristics to predetermined thresholds is how IS is determined. The method's usefulness is demonstrated by computational results in both IS and non-IS scenarios. It can even detect mains failure in situations where there is zero-power mismatch. Comparably, in^198^, a VMD-based method is put forth that computes the energy index using an equation that is empirically determined after breaking down the PCC voltage into four IMFs. Findings show that IS may be identified for loads with Q_*f*_ = 3.5, even at a signal-to-noise ratio of 20 dB. A unique technique for identifying IS occurrences is provided in^44^, whereby the SSKNN method and VMD are integrated. By extracting three-phase voltage signals, determining the major modes by VMD, and creating four feature indices based on the first three modes, this method works. Empirical findings validate the efficacy of this methodology, which conforms to the standards specified in IEEE 1547.

#### Kalman filter

An algorithmic mathematical tool called the KF is used to estimate variables over time intervals by taking into account noise and observed measures. It provides precise estimates of the state of a system by computing joint probability distributions across variables for every timeframe. It is appropriate for several applications, such as frequency measurements, harmonic decomposition, PQ monitoring, control of power conditioners and synchronization, and IS detection, due to its strong signal estimating capabilities, even in the face of distorted data. The KF has been used for loss of sources classification in the field of IS detection, as^43^ shows. An expanded version of the KF is used in this innovative IDM to estimate frequency in a MG context. The frequency variance is compared to a predetermined threshold to determine islanding. Based on computational results, it appears that the extended KF performs better in frequency estimation than both traditional KF and Fourier filtering. Additionally, even with balanced power settings, the method's accuracy in diagnosing IS seems promising; nevertheless, selectivity outcomes were not given.

#### Other signal processing based IDMs

TTT and PT are two more SP techniques used for IS identification. With PT, features are extracted from the input signal more effectively by using an adaptive measurement window and applying sinusoidal or co-sinusoidal functions to the sum of squared data samples. It is evident from^199^ and^200^ that PT is successful in detecting islanding. As opposed to this, TTT creates a two-dimensional representation of a one-dimensional signal using the ST. IS detection has made use of it in^201^.

## Recommendations and future trends

Based on thorough study and proven data, it is evident that each IDM has merits and demerits. Although active techniques offer a short NDZ and a quick detection speed, their impact on PQ might impact UGs performance. The islanding can be detected by passive methods and conventional security protocols. However, these systems might not identify the islanding condition when the DG rating is equivalent to the load power demand, leading to a large NDZ. On the other hand, remote methods provide excellent accuracy and detection speed and are compatible with large systems. However, installation costs, the stability of communication links, and computation speed are the primary drawbacks of remote ID systems. The recently published SP-based islanding identification methods make use of time and frequency domain-based SP to speed up detection and reduce NDZ. The effectiveness and success of these methods are also increasing when pattern recognition and AI techniques are applied. These systems have a large computational cost since there are several testing and training techniques, which makes them less desirable compared to other IDMs.

Taking into account the literature available for ID during the last 2 decades as well as recent technological breakthroughs, the future path of study with the viability of the practical application of IDMs is provided in the following.

1. The use of FPGA-based real-time implementation of complex digital SP algorithms might aid in the assessment of IS detection methods.
2. By incorporating components like PMU, smart meters, and other similar technologies, the quickly developing smart grid technology may be employed for IS detection. It will be feasible and profitable in this case since no additional hardware or software will be required for IS detection.
3. Recent technical developments open the door to the potential use of smart relays that use computational intelligence-based methods like ANN. Additionally, a multiprocessor with REOMP is capable of supporting the implementation of an ANN. Rapid FPGA technology permits a system-on-chip approach based on the implementation of REOMP that is flexible in its conceptual architecture.
4. By feeding the feature vector created by powerful digital SP algorithms into computational intelligence methods, ID accuracy will be further enhanced.
5. The most recent development in computational intelligence methods is DL, which replaces the need for feature vectors produced via SP methods with its capacity to extract features from raw signals. Such technology's real-time analysis can be very significant for ID.

Table 3 presents an overview of the primary performance metrics of the examined IDMs with respect to the NDZ outcomes, detection duration, power quality impact, multi-DG operation, suitability, X/R ratio reliance, and efficient functioning.

**Table 3:** Comparison taxonomy.

	Method	Reference	NDZ	Detection Time	Working with multiple DGs	Suitable for SSSG/SGBMG/IBMG	Dependence on X/R Ratio	Effective in weak grid /strong grid	Power Quality Impact
Remote	Impedance insertion	^16^	High	1.5 s	–	SGBMG & IBMG	Moderate	Weak grid	High
	DTT	^58^	Small	500 ms	Working satisfactory with 2 DGs	SGBMG & IBMG	Dependent	Effective in both	None
	PLCC	^57^	Small	200 ms	Working satisfactory with 2 DGs	SGBMG & IBMG	Moderate	Effective in Strong Grid	None
	SCADA	^62^	Small	–	–	SGBMG & IBMG	Moderate	Effective in Strong Grid	None
	PMU	^65^	Small	0.2–498 s	–	SGBMG & IBMG	Low to Moderate	Effective in Strong Grid	None
	MPMU	^72^	small	14 ms	Working satisfactory with 2 DGs	SGBMG & IBMG	Low to Moderate	Effective in Weak Grid	None
Passive	HD	^79^	High	53–129 ms	Working satisfactory with 3 DGs	SGBMG	Moderate to High	Effective in Strong Grid	None
	ROCOF	^88^	Medium	250 ms	Working satisfactory with 2 DGs	SGBMG	Low to Moderate	Effective in Strong Grid	None
	ROCOV	^105^	Small	510 ms	Working satisfactory with 2 DGs	SGBMG	Low to Moderate	Effective in Strong Grid	None
	ROCPAD	^110^	Small	100 ms	–	SGBMG	Moderate	Effective in Weak Grid	None
	ROCOP	^111^	Large	200 ms	Potentially affected	SGBMG	Low	Effective in Strong Grid	None
	ROCORP	^112^	Medium	–	–	SGBMG	Low	Effective in Weak Grid	None
	ROCOFOP	^113^	Large	300 ms	–	SGBMG	Low	Effective in Weak Grid	None
Active	AFD	^119^	Large	269 ms	unsatisfactory	IBMG	Moderate	Effective in strong grid	High
	IAFD	^115^	Large	200 ms	unsatisfactory	IBMG	Moderate	Effective in strong grid	Medium
	AFDPCF	^116^	Small	400 ms	unsatisfactory	IBMG	Moderate	Effective in weak grid	Low
	SFS	^119^	Small	167 ms	–	IBMG	Moderate	Effective in weak grid	Low
	SMS	^127^	Small	17–115 ms	–	IBMG	High	Effective in Strong Grid	Low
	APS	^126^	Small	200 ms	–	IBMG	High	Effective in Strong Grid	Low
	SVS	^130^	small	312 ms	–	IBMG	Moderate to High	Effective in Weak Grid	low
	RPV	^134^	Medium	200 ms–1 s	unsatisfactory	IBMG	Moderate to High	Effective in Weak Grid	Significant
Hybrid	SFS and frequency estimation based method	^29^	Medium	–	Working satisfactory with 2 DGs	SGBMG & IBMG	Moderate to High	Effective in Both Grid Types	Medium
	SMS and frequency estimation Based Method	^163^	Medium	–	–	SGBMG & IBMG	Moderate to High		small
	Voltage Unbalance and frequency perturbation	^30^	–	150 ms	Working satisfactory with 2 DGs	SGBMG & IBMG	Moderate to High		Medium
	ROCOV and active power perturbation	^106^	Medium	14 ms	Working satisfactory with 2 DGs	SGBMG & IBMG	Moderate to High		small
Machine Learning	DT	^168^	Small	30 ms	Working satisfactory with 2 DGs	SGBMG & IBMG	Low	Effective in Both Grid Types	None
	ANN	^41^	small	15 ms	–	SGBMG & IBMG	Low		None
	SVM	^178^	Medium	200 ms	Working satisfactory with 3 DGs	SGBMG & IBMG	Low		None
	ANFIS	^183^	Medium	500 ms	–	SGBMG & IBMG G	Low		None
	DL	^186^	Small	180 ms	–	SGBMG & IBMG	Low		None
Signal Processing	WT	^42^	small	300 ms	–	SSSG	Low	Effective in Both Grid Types	None
	MM	^193^	Small	20 ms	Working satisfactory with 3 DGs	SGBMG & IBMG	Low		None
	ST	^195^	Small	40 ms	Working satisfactory with 2 DGs	SGBMG & IBMG	Low		None
	HT	^196^	Medium	14 ms	–	SSSG	Low		None
	TTT	^201^	Medium	700 ms	Working satisfactory with 2 DGs	SBHMG	Low		None

## Conclusion

Based on their operational principles, the major IDMs were divided and discussed. Finally, a summary of the benefits and limitations of each strategy can be created, emphasizing the primary referenced studies that dealt with the issues associated with each IDM. The IDMs situated on the grid side or the DGs side make up the remote techniques. This approach's benefits include the avoidance of detrimental impacts on NDZ and power quality, and its efficacy in multi-DG islanding; its disadvantages, however, stem from its high implementation costs, higher complexity, and required maintenance. The passive methods comprised the second set of IDMs. The islanding diagnosis, which is determined by tracking the PCC variables, is carried out by comparing the collected data with predetermined cutoff points. This kind of IDM has low efficiency under power balance settings, despite its simplicity.

Following the evolutionary timeline, the active solutions introduce small disturbances into a certain electrical variable to increase the correctness of the passive procedures. These methods may cause the inverter output's component to fluctuate. The monitoring and disturbance injection phases make up hybrid methods. The previously indicated passive methods are generally used for monitoring in the cited works, while the disturbance injection is carried out using well-established active methods. Consequently, they outline the general advantages and drawbacks of both passive and aggressive methods. It is crucial to emphasize the ML algorithms' capacity for quick islanding identification in multi-DG situations as well as their discrimination for non-islanding occurrences. Nearly all of the ML based IDMs references that have been mentioned show the outcomes of non-islanding events such line to ground faults, motor starting, and capacitor bank switching. However, the quantity and quality of the data training set, as well as the number of extracted features, all affect how well those solutions function.

The SP-based methods make up the final category of techniques. These methods can generally be used for passive IDM, pulling pertinent data out of the sensed data. The results of the referenced literature show quick islanding identification and strong selectivity capabilities.

## Data Availability

The datasets used and/or analyzed during the current study available from the corresponding author on reasonable request.

## Abbreviations

MG: Microgrid
UG: Utility grid
NDZ: Non-detection zone
IS: Islanding
I_i_: Intentional islanding
U_i_: Unintentional islanding
T_D_: Detection time
IDM: Islanding detection methods
DERs: Distributed energy resources
EPS: Electrical power system
DG: Distributed generation
ML: Machine learning
DL: Deep learning
SP: Signal processing
PQ: Power quality
PCC: Point of common coupling
ANN: Artificial neural networks
FL: Fuzzy logic
SVM: Support vector machine
KF: Kalman filter
WT: Wavelet transform
CADM: Change of angle difference method
FDM: Frequency difference method
PMU: Phasor measurement unit
HI: Harmonic injection
ROCOF: Rate of change of frequency
SOGI: Second order general integrator
ROCOV: Rate of change of voltage
ROCOFoP: Rate of change of frequency over power
ROCOVoP: Rate of change of voltage over power
ANFIS: Adaptive neuro-fuzzy inference system
ROCPAD: Rate of change of phase angle difference
MPMU: Micro phasor measurement unit
PLCC: Power line carrier communication
SCADA: Supervisory control and data acquisition
OUV: Over/under voltage
OUF: Over/under frequency
MSVS: Modified Sandia voltage shift
HVDC: High voltage direct current
PWM: Pulse width modulation
THDv: Total harmonic distortion of voltage
THDi: Total harmonic distortion of current
SMS: Slip mode shift
SFS: Slip mode frequency shift
APS: Automatic phase shift
FT: Fourier transform
DFT: Discrete Fourier transform
WT: Wavelet transform
ST: Stockwell transform
PT: Phaselet transform
TTT: Time time transform
VMD: Variation mode decomposition
EMD: Empirical mode decomposition
I_i_: Intentional islanding
U_i_: Unintentional islanding
DTT: Direct transfer trip
DT: Decision trees
GA: Goertzel algorithm
ECS: External capacitor switching
PJD: Phase jump detection
HD: Harmonic distortion
ROCOP: Rate of change of active power
ROCORP: Rate of change of reactive power
VMD: Variational mode decomposition
IMF: Intrinsic mode function
PLL: Phase locked loop
ZCD: Zero cross detector
EMC: Empirical mode composition
HT: Hilbert transform
HHT: Hilbert–Huang transform
EMD: Empirical mode decomposition
ROCOFoP: Rate of change of frequency over power
RPV: Reactive power variation
C_*f*_: Chopping factor
APJPF: Active phase jump with positive feedback
APSO: Adaptive particle swarm optimization
RPS: Real power shift
KNN: K-nearest neighbors
FNN: Feedforward neural network
TQWT: Tunable Q factor wavelet transform
DNN: Deep neural network
SVD: Singular value decomposition
WSE: Wavelet singular entropy
WPT: Wavelet packet transform
MMRI: Mathematical morphology ratio index
DED: Dilation–Erosion differential
MIRF: Median-based islanding recognition factor
CIRFC: Current-based islanding recognition factor
RMS: Root mean square
SSKNN: Subspace-K-nearest neighbor
SGBMG: Synchronous generator based microgrid
IBMG: Inverter based microgrid
SSSG: Small scale synchronous generator
